# Advanced Microfluidic Vascularized Tissues as Platform for the Study of Human Diseases and Drug Development

**DOI:** 10.2174/1570159X20666220706112711

**Published:** 2023-03-08

**Authors:** Behnam Noorani, Luca Cucullo, Yeseul Ahn, Hossam Kadry, Aditya Bhalerao, Snehal Raut, Ehsan Nozohouri, Ekram Ahmed Chowdhury

**Affiliations:** 1 Department of Pharmaceutical Sciences, Jerry H. Hodge School of Pharmacy, Texas Tech University Health Sciences Center, Amarillo, TX, 79106, USA;; 2 Department of Biological and Biomedical Sciences, Oakland University, Rochester, MI 48309, USA;; 3 Department of Foundation Medical Studies, Oakland University, William Beaumont School of Medicine, 586 Pioneer Dr, Rochester, MI 48309, USA;; 4 Department of Pharmaceutical Sciences, School of Pharmacy and Pharmaceutical Sciences, The State University of New York at Buffalo, New York, USA

**Keywords:** Cell lines, microfluidic, drug development, pharmacokinetic, permeability, tissue, alternative, endothelium

## Abstract

The vascular system plays a critical role in human physiology and diseases. It is a complex subject to study using *in vitro* models due to its dynamic and three-dimensional microenvironment. Microfluidic technology has recently become a popular technology in various biological fields for its advantages in mimicking complex microenvironments to an extent not achievable by more conventional platforms. Microfluidic technologies can reproduce different vascular system-related structures and functions that can be utilized for drug development and human diseases studies. Herein, we first review the relevant structural and functional vascular biology systems of various organ systems and then the fabrication methods to reproduce these vascular districts. We provide a thorough review of the latest achievement in vascular organ-on-chip modeling specific to lung, heart, and the brain microvasculature for drug screening and the study of human disorders.

## 1 INTRODUCTION

The cost of drug development has increased dramatically during the last few decades. This cost increase results from a multifaceted problem: a) inaccuracies and reproducible issues of current preclinical drug evaluation models; b) the translational predictability of these models; c) scalability and cost-effectiveness. The result of these persistent burdens on drug development translates into a decreasing number of newly developed drugs [1]. The regular process in the pharmaceutical industry involves developing and screening many drug candidates followed by *in vivo* testing (preclinical models) of the most promising ones before moving to a clinical trial. Preclinical models are essentially used to evaluate the effects of drugs at the cellular, tissue, organ, and systemic levels. These animal models have the advantage of providing the complexity of tissue environments, and to a limited extent, several aspects of the disease pathology the drugs are expected to address [2].

On the other hand, *in vivo* studies have remarkable limitations. To improve the reproducibility of the experimental testing, we need to scarify the individual inter variability, which is a significant aspect of the human population and can greatly affect how a patient responds to drug treatments (including potential side effects). Unavoidable biological differences between the animal model of choice and human patients [2-4] are another major problem affecting several aspects of drug treatment, including efficacy, metabolism, and tolerance. In addition, the cost, time, and effort involved in running such extensive *in vivo* testing are high and potentially considered ethically controversial. Not surprisingly, most preclinical *in vivo* studies (80%) failed to show therapeutic outcomes consistent with corresponding clinical trial results. As an alternative to animal testing, a push to develop suitable *in-vitro* and tissue models has ensued for quite some time (and is still ongoing). These models are now widely adopted in preclinical studies.

*In vitro* two-dimensional (2D) cell culture models allow cells to grow in a tightly controlled environment to assure high reproducibility and easy analysis [5-7]. However, the major disadvantage of these platforms is their limited ability to replicate the complexity and intricacies of the tissue/organ they aim to reproduce, thus significantly reducing their relevance to human physiology. For example, the lack of a three-dimensional (3D) cellular microenvironment and cell-cell interaction with the surrounding cellular milieu that exists *in situ* can severely impact the cells' physiological and biological features *in vitro*, thus altering their response to drug testing [8-10].

Organ-on a chip is a new and more complex class of *in vitro* models recently developed [11]. The major improvement over the previous 2D platform featured in this new system consists of a cellular microenvironment that better recapitulates the cells' originating tissue environment and conditions, including 3D structures and dynamic flow [12]. These advanced models can reproduce organ-level functions for use in drug screening, drug discovery, personalized medicine, development of disease models, and basic research applications [13-16]. Besides, microfluidic technology is now a key proponent in enabling the engineering of *in vitro* human vascular models. This is crucial since the vasculature is a vital element overall and an essential characteristic of vascular organs, including the lung, heart, liver, kidney, brain, and skin [17-19]. The vasculature is one of the most critical systems in our body, which is vital to maintaining the homeostasis of tissues and organs, providing nutrients and oxygen to the whole body, removing toxins and metabolic waste, and facilitating many primary physiological processes, including regulation of pH and temperature [20]. Microvessels and capillaries located deep within the tissue of functioning organs connect circulating blood and organ parenchyma (cells and tissues in the specific organ microenvironment). Their functions are integrated dynamic systemic processes that react to changes in the local tissue microenvironment [21] and provide a biological barrier to protect the body from foreign agents [16]. Therefore, functional abnormalities of the vascular system can lead to dysregulation of the corresponding microenvironment and promote the onset of various diseases. Thus, understanding the physiology and function of vascular tissues in healthy and diseased models can facilitate the development of new and more effective therapeutic options to improve disease outcomes. To this purpose, advances in microfluidic techniques have made it possible to recreate quasi-physiological microvascular structures and function within a laboratory setting.

Owing to the microscale's unique properties, microfluidic systems offer several advantages compared to traditional platforms [14, 22, 23]. For instance, microfluidic devices enable precise spatiotemporal control of fluids in channels, enabling controlled shear stress generation for vascular tissue and allowing for various forms of mechanical stimulation, including the stretching of cells [19, 24]. In addition, the integration of hydrogels with live cells enables the re-creation of physiologically relevant 3D microenvironments [19, 23]. Besides, the dimensions of microfluidic channels can be constructed to be physiologically comparable with specific microstructures in the vascular system. In tissue engineering, vascularization has been emphasized to perfuse large-scale engineered tissue for *in vivo* transplantation and supply oxygen and nutrients to the cells. In contrast, microfluidic chip focuses on *in vitro* blood vessel models, which can be used for *ex-vivo* studies of vascular biology, pharmacological modeling, and drug testing and screening.

This review discusses the recent development of vascular-on-a-chip for drug development studies. After a summary of key aspects of vascular biology, we highlight microfluidic technology methods for *in vitro* vascular structure fabrication, including microvessel patterning and vasculogenesis/ angiogenesis-based models. Following, we discuss using these microfluidic organ-on-a-chip technologies to tackle the challenges faced in traditional *in vitro* models to replicate key facets of various organ systems, including Lung, Heart, and Brain microvasculature. Uses of microfluidic vascular tissue to model human vascular diseases are also included. Future challenges and directions are highlighted in the final section.

## 2 BIOLOGY AND STRUCTURE OF VESSELS IN BRIEF

**Fig. (1):**
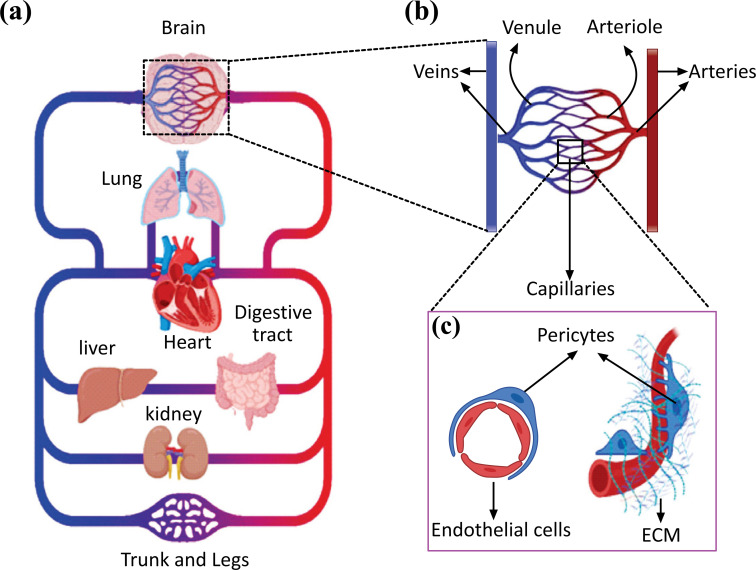
(**a**) Simplified illustration of the circulatory system (**b**) Schematic of arteries, arterioles, capillaries, venules, and veins in magnification. An artery divided into smaller arteries, arterioles, and capillaries. These drain into postcapillary venules, which collect into veins. (**c**) A schematic of capillary is shown in magnification, consisting of a single layer of endothelial cells supported by scattered pericytes and extra cellular matrix. Designed and created in BioRender application.

Pulmonary and systemic circulation constitutes the main branches of the human circulatory system, where the systemic circulation carries oxygenated blood from the left side of the heart to the body's tissues. The circulation system plays a critical role in providing oxygen and other nutrients to all body tissues while removing unnecessary metabolic byproducts (Fig. **1a**). The deoxygenated blood is then returned to the heart *via* a series of veins and passes through the pulmonary circulation, where it gets oxygenated and returned to the left heart. Based on their size and function, blood vessels can be classified into arteries (0.1 mm to >1 cm), arterioles (10-100 µm), capillaries (4-12 µm), venules (10-100 µm), and veins (0.1 mm to >1 cm) (Fig. **1b**). Large arteries and veins work as conducting vessels, and typically their vascular walls encompass three layers: the intima, media, and adventitia [25, 26]. The intima is the innermost layer formed by a thromboresistant confluent monolayer of endothelial cells (EC) attached to a basement membrane [25, 27]. A dense population of concentrically organized smooth muscle cells (SMCs) with bands or fibers of elastic tissues comprised the media. The adventitia, the outermost layer, is a collagenous matrix encompassing mainly fibroblasts and perivascular nerves [28]. Some of these layers might be less evident in microvessels, including arterioles, capillaries, and postcapillary venules [29]. Microvessel supplies oxygen and nutrients to tissues and exchange nutrients and waste products within tissues, but it also maintains tissue homeostasis through various biochemical pathways and functional barriers. Arterioles typically comprise one or two layers of smooth muscle cells (SMCs) and a thin, poorly defined adventitia. They control the regional perfusion of capillary beds through the contraction of circumferentially arranged vascular SMCs [29, 30]. Capillaries lack either a tunica media or adventitia. They are composed of a single layer of endothelial cells with an underlining basal lamina, surrounded by scattered pericytes that support and help maintain vascular integrity (Fig. **1c**) [31].

## 3 MICROFLUIDIC FABRICATION OF VASCULAR NETWORKS

Fabrication of vascular networks allowed for the development of 3D *in vitro* models and tissue-engineered devices as new research tools to further our understanding of human diseases and assess the efficacy, toxicity, pharmacokinetic and pharmacodynamic (PK/PD) profiles of new drugs for better therapeutic outcomes. Early work on developing flow-capable BBB models involved the use of 2D vascularized constructs of non-biodegradable materials, *e.g.*, silicon [32] and polydimethylsiloxane (PDMS) channels [33] using photolithography and soft lithography techniques. The two microfabricated polymeric cell-culture compartments in these constructs made up the tissue and blood sides [19, 33, 34]. Advances in biomaterials and microfabrication techniques brought to the development of three-dimensional microfluidic chambers and tubular, branching synthetic vessels with complex geometries perfused with media under continuous flow. Benefits of shear stress and co-culture to EC have been demonstrated with comparative studies in numerous 2D vascular barriers on chips indicating improved barrier and vascular functions. Ideally, substrates used for creating the platform should be clear, inert, nontoxic, implantable, and allow micropatterning with good fidelity at the submicrometer level [29]. Generally, microfluidic fabrication of vascular networks can be achieved following two main strategies according to their respective formation mechanisms: microvessel patterning and vasculogenesis/angiogenesis-based (self-assembled formation). Microvessel patterning engrave vascular-like microchannels directly into cell-laden hydrogel scaffolds *via* modified microfabrication techniques [19]. While vasculogenesis/angiogenesis-based technology involves the self-assembly of real vasculatures by using hydrogels embedded with ECs in microfluidic devices [19]. We will discuss both strategies in the following section.

### 3.1 Microvessel Patterning Techniques

**Fig. (2):**
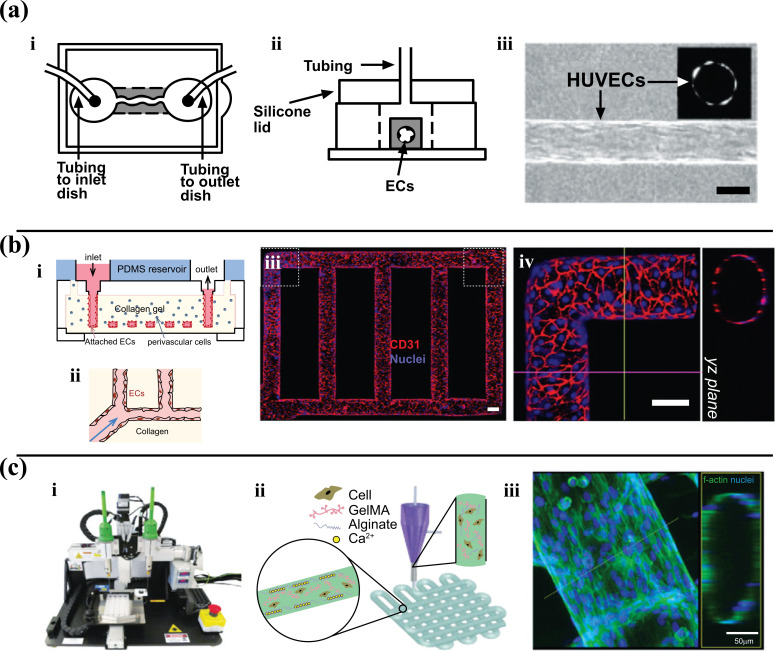
Microvessel patterning techniques. **a)** Needle-removal technique. A channel created by removing a needle from collagen gel. (i-ii) Schematic view of the chip and perfusion process. (iii) View of seeded endothelial cells in channel (scale bar is 100 μm) (Reproduced with permission from [36]). **b)** A microfluidic vessel networks manufactured by implementing micro molding technique accompanied by additive bonding and stacking of two collagen hydrogel slabs (i) Schematic cross-sectional perspective of the microfluidic collagen structure after fabrication (ii) Schematic of morphology and barrier function of endothelium in hydrogel. (iii) Z-stack projection for horizontal confocal sections of microfluidic vessels, (iv) view of a corner and YZ plane (Red: CD31, blue: nuclei. With a Scale bar: 100 μm) Adapted from Ref. [38] **c)** 3D bioprinting of microfibrous scaffolds for engineering of on-chip myocardium. (i) Photograph of the bioprinter. (ii) Schematic diagrams showing the crosslinking process of hydrogel (iii) Confocal fluorescence photographs depicting formation of endothelium and peripheral distribution of HUVECs. Replicated with permission from reference [47].

Microvessel patterning techniques involve the initial formation of empty cylinders seeded with cells. The Tien Laboratory developed these techniques. They utilized lithographic techniques to mold a silicone stamp with 1mm deep indentations. A 120 µm diameter stainless steel needle was embedded in collagen and later removed [35]. Endothelial cells were then introduced and formed a confluent layer with good barrier function (Fig. **2a**). As a follow-up to this study, the needle-removal technique was utilized in Khademhosseini Laboratory to examine the effects of fibroblast encapsulation within a hydrogel matrix [36]. Similarly, Yoshida *et al.* embedded silica tubes within a poly-c-glutamic acid matrix, removing them to form synthetic microvessels lined with a bilayer of SMCs and human umbilical vein endothelial cells (HUVECs) for assays of vascular permeability [37].

The above approaches can be reproduced quite efficiently but have limitations in generating the pattern and complexity of blood vessels *in vivo*. To produce a vascular network consistent with the complicated geometrical features of *in situ* blood vessels, a patterned PDMS stamp was used to engrave channels onto a collagen hydrogel and bond to a second flat collagen hydrogel layer. Based on (Fig. **2b**), this model became a small network of vessels that can be utilized for comprehensive studies of angiogenesis and vessel–blood interactions [38]. Bischel *et al.* fabricated branching synthetic vessels by forming a PDMS construct with hollow lumens filled with a liquid hydrogel polymer. The polymer was then washed away with the media flowing to the center [39]. The endothelial cells were seeded in the hollow lumens to study angiogenesis [39]. He *et al.* recently reported circular and branched vessels formed in enzymatically-crosslinked gelatin hydrogel by employing a master mold with predefined semi-circular channels recapitulating the *in vivo* structure of a vascular network [40]. Zhang *et al.* engineered vasculature within ultraviolet-polymerizable biodegradable scaffolds containing micro/nanopores, which were generated by mixing porogenic solvent into the hydrogels. This strategy allowed fabricating complex and porous 3D vascular networks by aligning pre-patterned scaffolds in an exceedingly layer-by-layer configuration [41].

Sacrificial molding techniques were also used successfully by other research groups to create complex vessels. In this process, a negative mold is built of dissolvable materials (as a template), embedded within a hydrogel structure, and then dissolved to leave behind hollow microvessels. The Tien Laboratory pioneered this technique by embedding a gelatin mesh within a collagen/fibrin matrix, with the mesh later dissolved by temperature changes [42]. The Miller Laboratory employed bioprinting to create a sacrificial carbohydrate lattice that dissolves in the aqueous cell culture medium, thereby leaving behind perfusable vascular networks that can be constructed in various natural and synthetic extracellular matrix (ECM) materials, including agarose, alginate, poly(ethylene glycol) (PEG)-based hydrogels, *etc.* [43]. Ultimately, the negative molding constructs determine the final geometry and the dimension of the *in vitro* microvasculature.

Another approach used to generate *in vitro* microvascular systems relies on 3D printing technology, generally referred to as the building of biological constructs in biomaterials and living cells using a computer-aided layer-by-layer deposition technique. This technology has been used to directly fabricate the vascularized networks of organ-on-chips [44, 45]. Notably, the recent advancements in multi-layered microfluidic coaxial extrusion systems have enabled the creation of complex 3D hollow channel arrangements in a simple and automated way. Incorporating the bioprinting technique into the development of microfluidic models brings more conveniences to researchers. Khademhosseini *et al.* extensively described the combination between the use of a tri-layer, double injection extrusion nozzle and a dual-stage cross-linkable bioink blend composed of gelatin-methacryloyl (GelMA), sodium alginate, and 4-arm poly (-ethylene glycol)-tetra-acrylate (PEGTA) [46]. The Cell-laden bioink was coaxially extruded between two calcium chloride (CaCl_2_) streams to facilitate the crosslinking function between alginate and Ca^2+^ on the outer surfaces of the initial tubular printed constructs, followed by covalent photocrosslinking of both GelMA and PEGTA components of the end-print [46]. Another study from this group showed bioprinted vascularized microfibrous scaffolds used to construct the endothelialized-myocardium-on-a-chip model (Fig. **2c**) [47].

Microvessel patterning techniques offer several advantages, including the feasibility of accurate patterning of vessels (which can immediately be perfused with media) and the geometry of resultant microvessels where the hemodynamic forces within these devices can be precisely determined to study the effect of hemodynamic forces on the exposed cells. However, some drawbacks remain unresolved, such as creating endothelialized capillaries (less than 30 um). One of the problems when developing small vessels *in vitro* is the difficulty of reaching a sufficient cell concentration *in situ* to establish a confluent cell layer. Multiple groups are now working to develop and optimize alternative methods/protocols to produce more complex microvascular networks, including more sophisticated bioprinting technologies to enable the use of multicellular reiterations and mechanical stimuli to achieve the desired goal.

### 3.2 Angiogenesis/Vasculogenesis-Based Techniques

As an alternative approach, several attempts have been studied to recapitulate the natural processes of angiogenesis and/or vasculogenesis within a laboratory setting to initiate synthetic microvessel formation [19]. The migration and development of different vascular cells into organized vascular structures are based on their responsiveness to various physical inputs and biochemical signals. Without proper stimulation, endothelial cells cultured in an isolated environment will grow into disorganized networks of cells [19, 48]. By applying appropriate physical cues and cellular signaling, angiogenesis/vasculogenesis-based techniques for microvessel formation seek to drive cultured vascular cells to form organized microvascular structures. *In vitro* formation of the vascular network by ECs on a culture dish was reported for the first time by Folkman *et al.* in the 1970s [49]. Later, an increasing number of studies have been devoted to fabricating a dense capillary-like vascular network by 3D culturing of ECs and other relevant cell types within biomaterials composed of protein-derived natural polymers such as collagen, fibrin, and gelatin on a microfluidic chip [23, 50, 51].

Raghavan *et al.* showed that physical stimuli could promote vasculogenesis in an organized fashion [52]. They created PDMS channels lined with endothelial cells embedded within a collagen matrix and stimulated blood vessel formation by adding growth factors [52]. The results indicated that vessels would be formed along with the provided channels [52]. Similarly, Nikkhah *et al.* combined HUVECs with a GelMA prepolymer to build linear constructs using photolithography [53]. The endothelial cells in these constructs were able to form primitive vascular structures. The study revealed that the variations in the height of the constructs affect endothelial cell morphogenesis. The pressure gradients created in the devices that mimic the physiological interstitial flow can also provide physical cues to induce the self-assembly of vessels [53]. Some studies also suggest that patterning of ECM components can promote cellular differentiation, migration, morphogenesis [48].

Angiogenesis-based techniques are used to induce the sprouting of new vessels from existing larger vessels by co-culturing multiple cell lines or stimulating the cells biochemically with growth factors [29, 54]. One common technique encourages the angiogenic formation of a capillary network between two larger endothelialized vessels initially created by microfabrication techniques. In a study by Yeon *et al.*, they successfully used this strategy to induce the formation of perfusable synthetic capillaries between two prevascularized microvessels, utilizing co-culture of HUVECs with fibroblast cells [55].

Using fibroblast cell co-culture within a prevascularized construct, Kim *et al.* developed a model to study angiogenesis and vasculogenesis, as shown in another study [56]. They fabricated a PDMS microfluidic device with five distinct channels and filled the central one with a collagen and fibrin matrix [56]. They observed spontaneous vasculogenesis by seeding HUVECs in the middle channel and lung fibroblasts in the outermost ones, which led to functional and perfusable vessels that invade the center channel [56]. They showed angiogenesis by seeding HUVECs on only one side of the center channel and fibroblasts only in the outermost channel on the opposite side, with sprouting vessels crossing the center channel [56]. It has also been shown that a combination of growth factors, such as fibroblast growth factor with VEGF, promotes angiogenesis compared with the use of a single growth factor [57]. Utilizing this approach, Kamm and co-workers have successfully developed a platform for the *in-situ* formation of microvascular networks employing the vasculogenesis process of embedded ECs. They also demonstrated its applications in studying the phenotypical transition of bone marrow mesenchymal stem Cells (BM-hMSCs) towards mural cells [58], breast cancer cell extravasation [59], visualization, and quantification of tumor cell extravasation dynamics [60], as well as interactions between neuronal networks and motor neuron diseases [61].

**Fig. (3):**
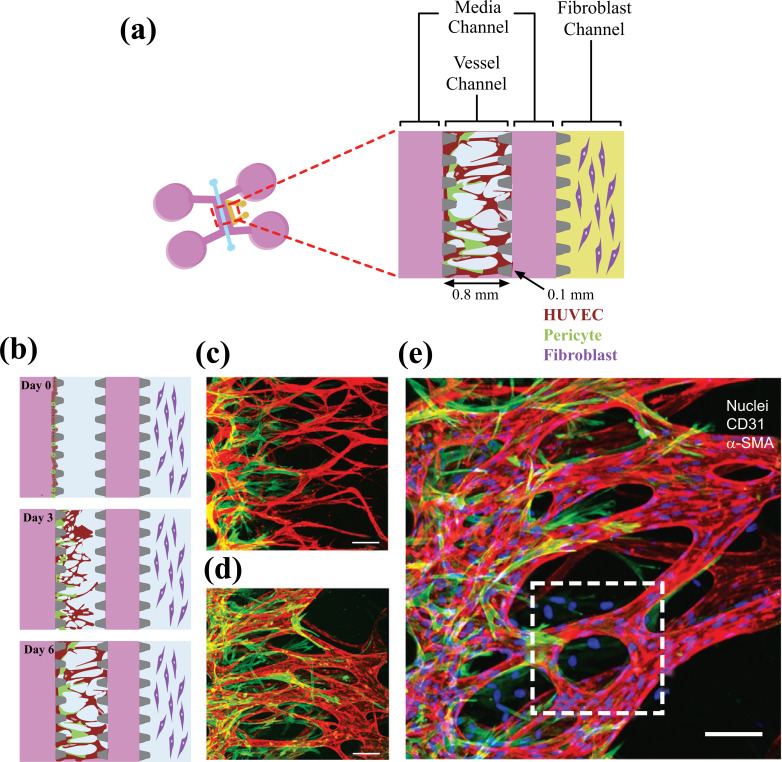
**a**) The microfluidic device is made up of a central vessel channel, two adjoining media channels, and the outermost fibroblast channel. The vascular network covered by the pericytes was structured in the central channel with the help of the lateral fibroblasts. **b**) The experimental plan of the progressive angiogenic process. Endothelial cells and pericytes were combined and attached to the left side of the vessel channel. Endothelial cells sprout through the fibrin gel to develop a blood vessel network, and pericytes followed behind the vessel. (**c**-**e**) Confocal images represent EC patterning in advance of pericyte association during the first 3 days (**c**), and matured pericytes covered the perfusable EC network on day 6 (**d**). Scale bars, 100 μm. Replicated (or adapted) with permission from reference [62].

Growth factors such as Vascular endothelial growth factor (VEGF) and basic fibroblast growth factor (BFGF) are utilized in the culture medium to spontaneously induce the capillary formation of endothelial cells in hydrogels. Even though this technique results in the successful capillary formation, the function or stability of the capillaries is not properly met. In these studies, regression of capillaries or leakage may result soon after their formation. Other types of cells proximal to ECs under physiological conditions, including pericytes, astrocytes, and dermal fibroblasts, were cocultured in hydrogels to develop a consistent vasculature with an improved endothelial barrier function [62]. Kim *et al.* developed a microfluidic platform that can be implemented to analyze the interactions between pericytes and ECs during the sprouting, growth, and maturation processes of neovessel formation. A combination of ECs and pericytes was attached to the side of a pre-patterned 3-D fibrin matrix, and it was allowed to sprout across the matrix (Fig. **3**).

In comparison to EC monoculture conditions, a remarkable reduction in diameter, a higher number of junctions and branches, and declined permeability were observed for EC-pericyte co-cultured vessels [62]. Angiogenesis/ vasculogenesis-based techniques are considered superior to microvessel patterning techniques as they allow for the formation of smaller and more complex vascular patterns with a branching level downsized to a few micrometers. Such models provide more convenient and reliable platforms to mimic the natural formation of native human microvascular systems that can be employed to examine the molecular mechanisms involved in vasculature formation phenomena and for studying vasculature-associated diseases. However, a few limitations still remain. These include the inability to create larger vessels and precisely control the pattern of vessel geometry. Furthermore, the formation of perfusable vessels is a lengthy process that may take many days to weeks to complete using angiogenesis/vasculogenesis techniques. In conclusion, the optimal method for synthetic microvessel formation depends on the vessel's intended use and may involve a combination of different techniques.

## 4 APPLICATION OF VASCULAR-ON- A-CHIP

Vascular microfluidic chips have been established to study human organ physiology in realistic conditions. These models can be used to study the involvement of blood vessels in the functional abnormality of the vascular organ, including lung, heart, and brain (Fig. **1a**). Besides, these vascular on a chip can investigate diseases related to the vascular wall, such as thrombosis and cancer metastasis. We discussed the use of these models in vasculature-related organ regeneration and the potential challenges. We then examined various microfluidic models of vascular diseases.

### 4.1 Vascularized Organs on a Chip

Several Organ-on-a-chips have been developed to mimic specific characteristics of human organs. However, as blood vessels closely interact with other organ systems (Fig. **1a**), fabricating vascular *in vitro* organ models such as lung, heart, liver, kidney, brain, and skin can significantly advance our understanding of organs physiology in disease and health conditions and improve our ability to predict drugs’ effects in the preclinical stage of development. The following paragraphs summarize recent efforts to establish vascularized *in vitro* organ models on a chip and their applications.

#### 4.1.1 Lung

The lungs are the primary organ conducting the respiratory function in humans. The lungs are part of the respiratory system composed of airways, multiple branched blood vessels, and two main zones. The conducting zone provides the in and out air routes, while the respiratory zone is where oxygen and carbon dioxide exchange occurs [63]. The parts of the conducting zone include the larynx, trachea, primary bronchi, secondary bronchi, and bronchioles, and the respiratory zone begins from the terminal bronchioles where they meet the respiratory bronchioles and then is connected to the clusters of alveoli [63]. All those parts contain epithelial cells and a basement membrane. Two types of alveoli exist Type I (AT1) and Type II (AT2). AT1 cells are flattened and squamous, comprising more than 95% of the gas exchange surface of the lung, and intimately associated with the underlying endothelial capillary plexus to form the thin gas diffusible interface. AT2 cells are cuboidal and produce pulmonary surfactants to prevent alveolar collapse during respiration [63]. The alveolar sac is a cluster of alveoli, and an alveolus is a single functional unit of the lung. An alveolus can expand its wall with the incoming air to provide a large surface area for efficient gas exchange. The major and unique challenge for developing the *in-vitro* biomimetics of the lungs is the generation of the mechanical forces that periodically and dynamically change with the respiratory cycle. The respiratory system demonstrated the dynamic behavior of cyclic motion physical stresses, as the lung is fundamentally a mechanical organ. The lungs experience a considerable change in volume with each breath and an approximate alteration in tissue length of 4%-25% [64]. Therefore, the reproduction of lung structures and functions *in-vitro* is critical to comprehending the principle of infectious lung diseases and the impacts of the xenobiotics such as drugs and toxins. Microfluidic chips were implemented to create an *in-vitro* model of healthy and infected lungs with proper fluid flow and sustained gas exchange.

Researchers have recently focused on lung-on-a-chip (LOC) models to reproduce mechanical forces, including the air pressure and the effects of shear stress and their impact on the progression of respiratory diseases. A pioneering LOC model was developed using soft lithography to separate the chip into regions by 10 μm PDMS membranes filled with an extracellular matrix [65]. Alveolar epithelial cells were seeded in the upper PDMS regions, and human pulmonary microvascular endothelial cells were cultured in the lower regions, replicating the geography of the alveolar-capillary barrier [65]. The mechanical stretches and elastic recoils of the PDMS membrane were produced under a vacuum condition to mimic the expansion and contraction of the alveoli [65]. With this setup, endothelial cell alignment responses mimicked those of the actual endothelium and *in vivo* blood vessels [66], differing from cell behavior in 3D culture systems that do not incorporate a mechanical actuation [67]. A medium containing the proinflammatory cytokine tumor necrosis factor α (TNFα) was introduced into the LOC’s vascular channel to create an inflammatory condition. The inflammatory response was monitored by measuring the expression of intercellular adhesion molecule 1 (ICAM-1) and subsequent adhesion and transmembrane migration of fluorescently labeled neutrophils [65]. This LOC was the first demonstration of a mechanically active system generating more realistic *in vitro* lung environments and subsequent immune responses and has since become a foundation in the microfluidic configuration for further development of LOC devices.

Building on the micro-engineered system that Huh *et al.* pioneered, Stucki and colleagues developed a 3-D lung chip that contains an alveolar barrier and cyclic strain to reproduce the expansion of the alveolar walls [68]. A reproducible 3D cyclic strain was used to mimic the movements of the diaphragm. This model was the first successful *in-vitro* design to show the movement of the alveolar wall using an elastic membrane to simulate respiration [68]. The study showed that mechanical stress affected the permeability properties of the epithelial barrier [68].

Another 3-D lung chip was developed by Blume *et al.* to simulate the pulmonary interstitial fluid exchange by allowing the flow through the cell culture system of the airways [69]. This model provided a better understanding of the function of the epithelial barrier in the lungs. A stent with a permeable filter was used as a single tissue culture chamber to improve the model's integrity. Altogether, the realistic simulation of the lung environment was achieved by enabling the exposure to shear flow and pressure to the alveoli (and the attached capillaries) and by using an elastic membrane to mimic the dynamic change of the interface of the pulmonary cells during the respiratory dilation [69].

The pulmonary structural features between the epithelial and endothelial cells were studied by Benam *et al.* using the microfluidic chip model that creates a tissue-tissue interface [70]. The device consisted of two parallel PDMS channels separated by porous phosphatidylserine (PS) membrane coated with type I collagen. The top channel where the air goes in and out was seeded with epithelial cells, while the bottom channel contained vascular endothelial cells exposed to media flow. These channels make up the barriers between the two cell types and maintain a highly stable condition over several weeks [70]. This microfluidic model was utilized to mimic the pathophysiology of asthma and chronic obstructive pulmonary disease (COPD) and examine the effect of interleukins (IL)-13 treatment. To verify the disease model, Benam and colleagues measured the level of IL-8, RANTES, cellular damage, and goblet cells, which are usually found in asthmatic lungs. Also, the following treatment of rheumatoid arthritis with tofacitinib was tested on the model to inhibit the signaling pathway activated by IL-13 or dexamethasone. The results were similar to previous clinical outcomes; tofacitinib, which explicitly inhibits Janus kinases (JAK), thus blocking cytokine receptors’ activity, reverses cilia functions, and decreases cytokine levels. By contrast, corticosteroids did not show any therapeutic effects [70].

**Fig. (4):**
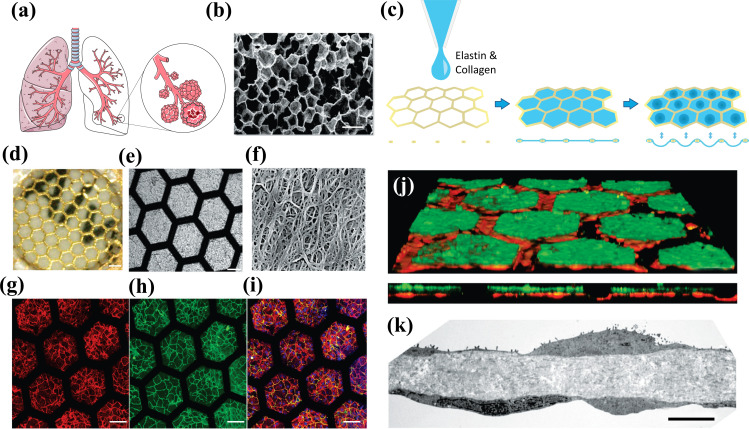
Fabrication of the lung alveoli array on a chip: **a)** a Schematic of the respiratory tree-like structure ending with alveolar **b)** Scan electron microscope of a slice of human lung parenchyma with tiny lung alveoli and their ultrathin air–blood barrier (Scale bar: 500 µm) **c)** Schematic of the production of the collagen–elastin (CE) membrane used in the generation lung-on-a-chip. A thin gold mesh with an array of hexagonal pores of about 260 µm is used as a scaffold, on which a drop of collagen–elastin solution is pipetted. The collagen–elastin gel forms a suspended thin membrane that can be stretched at the alveolar level by applying a negative pressure on the basolateral side of the membrane. **d)** a Optical clarity of a 10-µm-thin CE membrane integrated in the gold mesh. (Scale bar: 200 µm) **e)** Picture of an array of several hexagons with a CE membrane (Scale bar:100 μm). **f)** SEM picture of the collagen and elastin fibers of the CE membrane (Scale bar: 500 nm). **g-i)** Immunostaining of primary human lung alveolar epithelial cells (hAEpC). Expression of adherent junction markers E-Cadherin, red), tight junctions with zonula occludens-1 (ZO-1, green) and merged (Hoechst, blue; E-Cadherin, red; ZO-1, green) after two days interfence with air (Scale bar: 100 μm) **j**, **k)** Air–blood barrier reproduction. J) Confocal pictures (perspective view and cross-section) of a coculture of hAEpC (E-Cadherin in green) with human primary endothelial cells (Rfp-label in red) on the hexagonal mesh with the CE membrane. (Scale bar: 100 μm). b TEM picture of hAEpC type I-like cells in coculture with human lung endothelial cells at day 4. (Scale bar: 5 μm) Replicated with permission from reference [71].

Zamprogno *et al.* fabricated a lung-on-a-chip based on a biological, stretchable, biodegradable membrane made of collagen and elastin that mimic an array of tiny alveoli with *in vivo*-like dimensions (Fig. **4**) [71]. The air-blood barrier was formed using primary lung alveolar epithelial cells from patients and primary lung endothelial cells. The barrier properties were preserved for up to 3 weeks with typical alveolar epithelial cell markers expression. The morphology of type I (ATI) and type II (ATII) lung alveolar epithelial cells were discernible by transmission electron microscopy (TEM) imaging. Tight junctions, a further characteristic of lung alveolar epithelial cells, were evidently expressed along the cell borders at day 4 and day 21 [71] and the permeability of the CE membrane with a monolayer of lung alveolar epithelial cells was assessed with two paracellular markers (FITC–sodium, FITC–dextran) [71].

Xu *et al.* developed a pulmonary cancer model using a microfluidic chip to replicate the microenvironment of lung cancer [72]. In this study, a microfluidic device was created using a nanofiber membrane made from polylactic-co-glycolic acid (PLGA) and PDMS, which was made to be biologically compatible, porous, and permeable. Cell viability and cytotoxicity were tested in this device after treatment with the anti-cancer drug gefitinib. Human nonsmall-cell lung cancer cells (A549), human fetal lung fibroblasts (HFL1), and HUVECs were used for the study. A549 cells alone showed sensitivity to the drug, but the co-culture system with HFL1 increased the cell viability and survival rate of the cancer cells even when treated with gefitinib. When all the three cell lines were cultured together, the invasion of tumor cells prompted by A549 led HUVECs to death. This lung-on-a-chip platform effectively reproduced the physicochemical characteristics of the lung environment and successfully predicted the therapeutic outcomes of the tested drugs [72].

One of the most complex microfluidic orthotopic lung tumor models describes the effect of breathing on tumor growth and consequent vascular invasion [73]. Human lung cancer cells that express high levels of fluorescent proteins for monitoring tumor growth were injected into the epithelial tissue layer channel. Upon exposing the chip to cyclic mechanical stretching (simulating the mechanical cues of breathing), the tumor cells grew in the center of a small area of epithelium instead of spreading over a larger area, and their proliferation rate decreased by 50% [73]. The results from this work provided evidence for a positive motion-dependent feedback loop where the mechanical strain associated with unencumbered breathing decreases the phosphorylation of epithelial growth factor receptor (EGFR). In contrast, the loss of motion from increased cell proliferation in the alveoli increases tumor growth and EGFR phosphorylation [73].

Recently, the cytotoxicity of ZnO and TiO2 was assessed using an *in-vitro* pulmonary chip model. The investigators measured the reactive oxygen stress level and apoptosis of the epithelial and endothelial cells, followed by ZnO and TiO2 treatments. The device was incorporated into two parallel and separated channels; a lower chamber seeded with human umbilical endothelial cells, and the upper channel was cultured with human pulmonary alveolar cells. The central channel covered with the matrigel was intended to mimic a basal membrane. The nanoparticles (NPs) were infused through the epithelial channel to simulate acute pulmonary nanoparticle exposure. The results showed that both ZnO and TiO2 particles induce apoptosis of the two cell lines in a dose-dependent manner. ZnO particles demonstrated greater membrane permeability resulting from a higher level of reactive oxygen species, which damaged the cell membranes [74].

Despite the many advances in microfluidic chip technologies, there are still many challenges that are yet unresolved. The endothelial channel in the alveolar chip, which aims at reproducing the pulmonary capillaries, is still significantly larger than a real alveolar capillary. The size difference impacts several physiological parameters, including shear stress, flow velocity, and the interactions/adhesion between the vascular endothelium and circulating immune cells (such as neutrophils) and tumor cells. Technical advancements to increase the cell seeding density will help develop LOC systems featuring more realistic (size-wise) embedded vascular beds, thus further improving these models' accuracy and translational relevance.

#### 4.1.2 Heart

The heart plays a crucial role as a biological pump to circulate the blood through the vascular systems across the whole body [75]. The heart is a complex organ of muscle tissue and blood vessels. Cardiomyocytes (CM) play a critical role in cardiac muscle contraction and are the main component of cardiac muscle tissue linked to each other by intercalated discs. Cardiovascular diseases (CVDs) are among the most significant leading cause of premature death in developed countries [75]. Hence, there is a strong demand for new drugs to prevent or treat CVDs; however, one of the main factors hindering CVDs drug development is the lack of models to mimic the heart tissue and its physiological responses adequately. On this front, while recent progress in microfluidic technology provides a better microenvironment for the study of heart diseases, several significant challenges are still unmet. The heart is a complex tissue and fabricating a platform capable of reproducing the hierarchical structure and function of the native myocardium (including mechanical contractions, electrical activity, molecular transport, and specific responses to drug stimulation) is still out of reach. Not surprisingly, the currently available state of the heart-on-chip (HoC) models is still unable to accurately mimic the complex microenvironment of the cardiac tissue [14]. In the heart, the phases of contraction and relaxation are repeated to produce the heartbeats, which causes the continuous deformations of cardiomyocytes. The continuous cell deformations generate repeated stress throughout the fibers of the ECM [76], which activates stress-sensitive signaling pathways affecting various physical and functional aspects of cardiomyocytes, such as orientation cell polarization and cell communication [76-78]. Indeed, the 3-D structure of the tissue and the electrical and mechanical cues of the cardiac system need to be integrated into the HoC models to improve their translational relevance. The section below discussed some of the current state-of-the-art HoC models and possible directions for improvements.

A 2-dimensional microfluidic HoC model was generated using cardiomyocytes derived from stem cells in the early stage of development. This model was able to maintain cell viability and functionality for several weeks for research studies in pharmacology. However, the predictability of such systems is limited due to their incapability in the recapitulation of human *in vivo* structure [79]. Later, Agarwal *et al.* successfully incorporated temperature control and electrical stimulation systems within a polycarbonate (PC) and aluminum microfluidic device and were able to observe an increase in cardiac contractility (both rate and strength) in response to isoproterenol treatment [80].

A three-dimensional model of human pluripotent stem cell-derived cardiac tissue was developed by Thavandiran [81]. This HoC platform was used to generate a tachycardic model of arrhythmogenesis based on cardiac microwires [81]. The system takes maximum advantage of mechanical stress for better assembly, maturation, and functionality of the cardiac tissue. At the same time, the small-sized model does not require vascularization [81]. Collagens and point electrodes enable the self-assembly of tissues in a 3D environment and stimulate the tissues electromechanically, respectively [81]. The resulting organ-on-chip model is better suited to mimic the development and propagation of electrical signals through the heart tissue but lacks the microfluidic environment necessary to carry out most pharmacological testing.

Alternative to this platform, another model was developed using PDMS to create the HoC structure. Specifically, the PDMS-based chip was coated with a collagen-chitosan hydrogel encapsulated by Thymosin beta-4 (Tβ4, a small peptide with G actin-sequestering action associated with induction of angiogenesis and accelerated wound healing) for culturing mouse veins and arteries. The micropatterns of PDMS assist the formation of the vascular structures, and Tβ4 encapsulating the hydrogel provides cardiac protection and promotes angiogenesis [82]. The vascularized model successfully achieved levels of excitation threshold, sarcomere organization, and cell-cell junctions more closely comparable to the heart *in-vivo* than the previous HoC platforms lacking the vasculature system [82].

Another microfluidic platform was recently engineered to reproduce the heart's mechanical forces generated by myocardial cells [83]. A PDMS membrane separates the two compartmentalized PDMS microchambers in this model. Cardiomyocytes and human iPSC-derived cardiomyocytes are seeded into a matrix of fibrin gel in the central channel and the culture medium filled across the side channels. The PDMS membrane is deformed by pressurizing the bottom compartment. In this manner, the 3D cell constructs are compressed. The process is cyclical to simulated systolic and diastolic phases [83]. The cultured cells in the 3-D platform exhibited steady and simultaneous movements like heartbeats in response to the electric pacing signals generated by electrodes incorporated in the platform [83]. The system was shown to respond to low concentrations of isoprenaline, thus replicating the chronotropic effect previously observed in hESC-derived heart tissue [84, 85] and iPSC-derived cardiomyocyte monolayers at higher doses [86].

More recently, HoC models have been seen incorporating additional features of tissue engineering and nanotechnologies. MacQueen *et al.* utilized polycaprolactone/gelatin nanofibrous 3D substrates to promote myocardial tissue assembly [87]. The heart ventricle ECM-inspired scaffold was placed inside a microfluidic bioreactor that included intra- and extraventricular flow loops and access ports for catheters [87]. The scaffold was seeded with both neonatal rat ventricular myocytes and human iPSC-derived cardiomyocytes. After 3-5 days of cell seeding, the engineered ventricle system stably reproduces the repeated and differentiated excitation patterns and synchronous contractions when stimulated with electrodes at 4-5 Hz [87]. The results support the model’s feasibility of mimicking arrhythmogenic heart diseases [88].

An anisotropic ventricular myocardium was also engineered by seeding myocytes into a combination of poly (N-isopropyl acrylamide), fibronectin, and PDMS micro construct patterns on a glass substrate. Despite the absence of microfluidics and 3D structures, the ventricular system assessed the cell contractibility, cytoskeletal architecture, and action potential propagations. Indeed, the model responded to epinephrine treatment showing chronotropic effects similar to that observed *in-vivo* [88]. Using hiPSC-derived endothelial cells and CMs from a single-cell source, Ellis *et al.* developed an even more realistic heart model. The endothelial cells were seeded on the entire space of the microvasculature channel, and the CMs were cultured in a central channel within GelMA pre-activated by UV light. The nutrient-rich media is continuously exchanged through the 200um gap between the channels. This co-culture system was shown to promote the hiPSC-CM maturation and better reproduce the organ complexity *in vivo* [89, 90], where the staggered microposts promote anisotropy and can affect cardiac function and cell growth [90]. The cardiac tissue in the system reached functional and biological maturation in two weeks of cell culture, exhibiting simultaneous contractions, complete sarcomere structure, and upregulated expression of HCN1 and KCNQ1 observed in the mature CMs cell line [90]. This more advanced system has already been used to generate a disease model of cardiac fibrosis [91]. In this study, co-cultured fibroblasts and hiPSC-CMs on a microfabricated device were exposed to TGF-β to trigger typical fibrosis followed by heart failure. Anti-fibrotic drugs were used to reverse the TGF-β effect, demonstrating the model's usefulness as a drug discovery and testing tool for cardiac fibrosis [91].

Unfortunately, despite the current advancement in the field, none of the current HoC models have succeeded yet in recapitulating the full complexity of the natural heart but only a specific set of properties and features. One of the significant challenges yet to tackle concerns the ECM environment, which is dynamic and not static. Furthermore, standardized techniques, protocols, and materials required to manufacture and establish these models must be put in place to allow for larger-scale manufacturing and distribution. Currently, microfluidic technology in laboratory settings has been confined to a limited number of investigators and research groups with little ability to transfer the technology across the field. This indeed limits the ability to validate these models' feasibility, reproducibility, and accuracy and attract private sector investment.

#### 4.1.3 The Blood-Brain Barrier

The blood-brain barrier (BBB) is a highly organized and selective vascular interface that regulates the transport of different molecules in and out of the brain [5]. BBB plays a vital function in protecting the CNS from undesired endogenous or exogenous molecules and maintaining the homeostasis of the brain environment for optimal neuronal activity [92]. The BBB provides a physical and multifunctional barrier to the brain environment by limiting molecules' passive transport (*e.g.*, interendothelial diffusion, pinocytotic activity, *etc.*) and strictly regulating the active transport systems [92]. The dysfunction and breakdown of the BBB contribute to multiple neurological disorders, including Alzheimer’s disease, epilepsy, stroke, multiple sclerosis, and traumatic brain injury [92]. The BBB dysfunction results in the passage of harmful blood components into the brain, uncontrolled transport, and dysregulated clearance of metabolites associated with reduced cerebral blood flow. It is still debatable in several diseases whether the pathological conditions result from a disruption in the BBB or whether the disruption of the BBB is the result of the disease condition (*e.g.*, epilepsy) [5]. Preclinical studies have shown BBB leakage, varying in different circumstances from widespread leakage to localized small leaks in other brain regions [92]. Therefore, there is a high demand for advanced *in-vitro* models to understand the physiology and mechanism of BBB impairment in a vast range of neurological, neurodegenerative and neuroinflammatory disorders. With the latest advances in microfluidic and nanofabrication technologies, developing *in vitro* BBB- and brain on-chip platforms that mimic the *in vivo* brain vascular microenvironment is becoming more feasible. Herein, we first discuss and reviewed current BBB-on-a-chip platforms, then we describe microfluidic models of neurological disorders.

The earliest models of BBB-on-chip originated from the Transwell platform, which from a practical standpoint, is a vertical, top to bottom diffusion system across a semipermeable membrane that provides support for culturing cells on both sides. Specifically, the brain and blood sections of the platform are built by vertically sandwiching a microporous semipermeable membrane in-between two microfabricated polymeric cell-culture compartments [11]. Fluorescent tracers can be used to assess the permeability, and real-time measurement of Transendothelial electrical resistance (TEER) can be performed with custom-designed electrodes [11]. Different comparative studies in multiple BBB-on-chip models have shown beneficial effects of applying shear stress to the endothelial layer and/or adopting a co-culture setting (astrocytes with or without pericytes) juxtapose to brain microvascular endothelial cells (BMECs) to improve the barrier integrity and functional viability [93-96]. For example, a 3D self-organized microvascular model of the human BBB with endothelial cells, pericytes, and astrocytes demonstrated improved barrier integrity and reduced permeability to paracellular markers of different molecular weights such as 10 and 40 kDa FITC-dextrans [97]. Another group showed similar results when primary human pericytes and astrocytes were co-cultured on the chip juxtaposed to the endothelial layer. In this case, the permeability of 3kDa FITC-Dextran decreased 3-fold compared to the endothelial monolayer alone [98].

Introducing microneedles or perfusion through an ECM-gel can form cylindrical microchannels, which help in emulating the circular cross-section of native vessels [99-103]. These BBB chips achieve a uniformly distributed flow profile and develop a natural basement membrane as BECs secrete their basal laminin. But the lumens in such BBB chips are still significantly larger (>100 um) than those of brain capillaries (7-10 µm). For example, Kim *et al.* developed a collagen-based 3D model of brain vasculature, shown in Fig. (**5a**). They used microneedles (250-350um) to create 3D tubes in the collagen I gel. After the microneedles were removed, the 3D microvessel was seeded with endothelial cells from the bEnd.3 to replicate the BBB. They found the microvessel was formed in hydrogel and the permeability results of 40kDa showed an intact barrier after 7 days [103]. Linville *et al.* used induced pluripotent stem cells (iPSC)-derived vascular endothelial cells to create a 3D human BBB model by seeding the endothelial cells into a microstructured channel of collagen hydrogel Fig. (**5b**). The vascular endothelial cells seeded in the channel developed a uniform monolayer with evident endothelial tight junctions’ proteins within six days. Efflux transporters' expression as immune activation was confirmed using a P glycoprotein (P-gp) inhibitor and cytokine activation, respectively. BBB opening by exposure to a high concentration of mannitol and the parallel measurements of BBB permeability to paracellular markers were used to assess the functional response of the barrier to hyperosmotic stimuli [100].

**Fig. (5):**
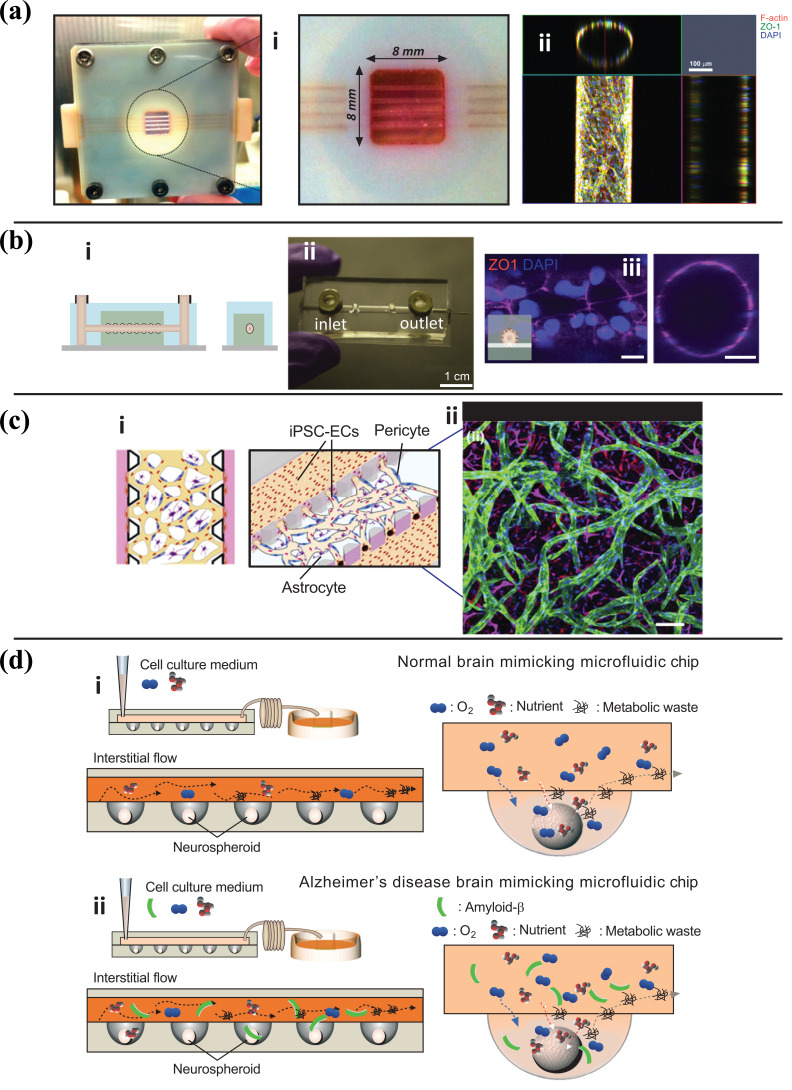
(**a**-**c**) BBB on chips with different methods of fabrication. **a**) (i) Post-fabrication photograph of brain microvasculature system with a magnified perspective of collagen microchannels. (ii) Confocal fluorescence micrographs showing a top view of the microvasculature array and an orthogonal view of the cylindrical lumen. Red: F-actin, green: ZO-1, and blue: nucleus. Reproduced with permission from reference [103] **b)** (i) Schematic illustrations of the side- and end-view of microvessel model. (ii) PDMS based microfluidic chip (iii) Fluorescence images of blood-brain barrier microvessels on day two ZO-1:Red, Nuclei: DAPI (blue). Reproduced with permission from reference [100]. **c**) (i) Schematic depiction of the three-dimensional BBB microvascular network through vasculogenesis which mimics the microvascular structure present in the brain environment. (ii) Confocal image of self-assembled BBB model, including iPSC-Endothelial cells (CD31, green), Pericytes (F-actin, red), and Astrocytes (GFAP, magenta), and nuclei (DAPI, blue). Reproduced with permission from reference. **d**) (i) Schematic diagrams of normal brain mimicking microfluidic chip and (ii) Alzheimer's disease brain mimicking microfluidic chip. (i) Neurospheroids on normal brain mimicking microfluidic chip were cultured under dynamic conditions with a flow of normal medium including oxygen and nutrient for 10 days. (ii) Neurospheroids on Alzheimer's disease brain mimicking microfluidic chip are cultured under dynamic conditions with a flow of normal medium including oxygen and nutrient for 7 days and then incubated with a medium including 5 μM synthetic amyloid-β. Reproduced with permission from reference [108].

The vasculogenesis strategy is based on the self-assembly of vascular cells. Upon loading cell-laden hydrogel into a microdevice, endothelial cells self-assemble into a vascular network, with pericytes and astrocytic endfeet directly attached to the surface (Fig. **5c**). The vasculogenesis based BBB-chips mimicking the hierarchical branching patterns *in situ* and having a lumen diameter of less than 50 µm are considered the model more closely resembling brain capillaries *in vivo* [97, 104]. However, due to the inherent heterogeneity in branching patterns, optimization is still essential to assure reproducibility.

**Fig. (6):**
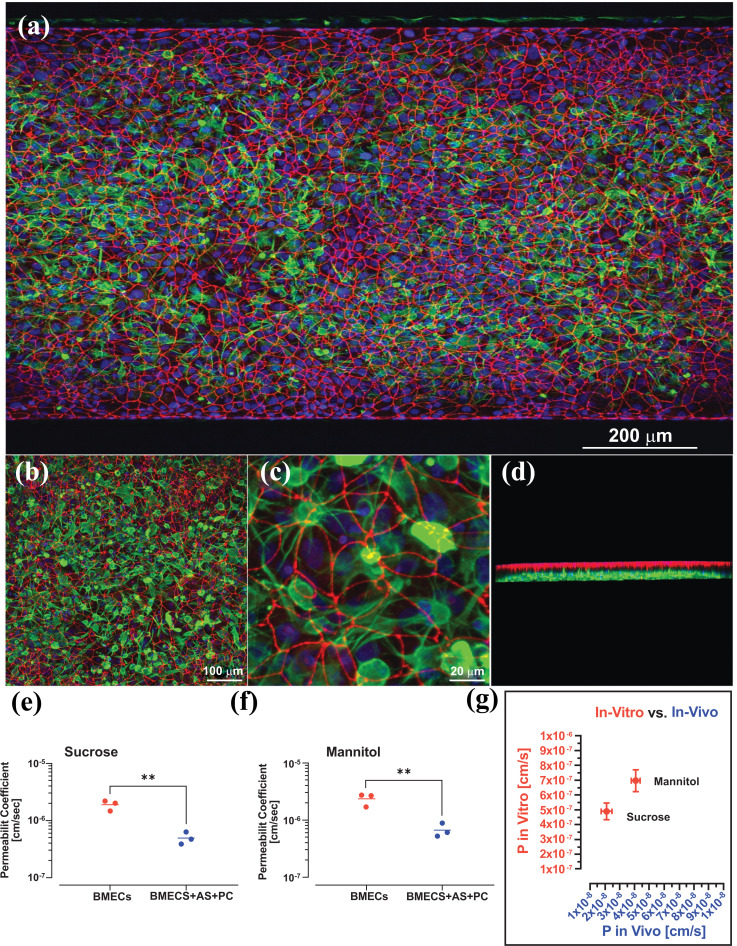
Immunofluorescence images demonstrating the co-culture of the BBB-on-a-chip. iBMECs cultured on all surfaces of the blood channel, and primary human brain astrocytes on the apical brain channel. **a**-**c**) different magnification of iBMECs stained with ZO-1(red) form a tight barrier function on the blood side and astrocyte stained with GFAP (green). **d**) 3D structure of co-culture. Comparing the permeability coefficient of paracellular markers **e**) sucrose, **f**) mannitol in BBB-on-a-chip with iBMECs alone and iBMECs with primary human astrocytes and pericytes cultured under continuous flow. Student’s t-test (p < 0.01); n = 3 Biological replicates. (**g**) Comparison between sucrose and mannitol permeability assessed in BBB microfluidic model with previously reported *in-vivo* data in mice (n = 3 biological replicates. Reproduced with permission from reference [106].

Stem cells derived from neurovascular cells offer potential avenues for advanced neurotherapeutics. Pluripotent and adult stem cells have the ability to self-renew and can be differentiated into a specialized cell phenotype or genotype, which can be of great help in developing drug screening models. Wang *et al.* utilized human induced-Pluripotent Stem Cells (iPSCs) derived BMECs (brain microvascular endothelial cells) to design an *in vivo* like BBB model with pump-less microfluidic compartments [105]. This gravity-based model achieved and maintained quasi-physiological high TEER values for up to 10 days [105]. Noorani *et al*., developed an advanced BBB microfluidic chip containing BMECs, human primary astrocytes and pericytes (Fig. **6**) [106]. Passive markers such as sucrose, and mannitol, were used to confirm the barrier function of the model. The presence of human pericytes and astrocytes significantly decreased the blood-to-brain paracellular leakage of sucrose and mannitol. Therefore, triculture with primary human astrocytes and pericytes increased the barrier function of the BBB on a chip (Fig. **6e**, **f**). Moreover, the barrier function of the *in vitro* BBB microfluidic chip were only 1 log magnitude higher than *in vivo* mice model [106].

A more complex BBB chip has been recently optimized to grow iPSC-derived BMECs, human primary astrocytes, and vascular pericytes, which showed quasi-physiological barrier functions, including low paracellular permeability and efflux activity, and osmotic responses [107]. Similar advanced platforms can also be used to evaluate drugs' efficacy and assess antibody penetration across the BBB, as recently shown by Park *et al.* [107]. Noteworthy is that a BBB chip can be fabricated using patient-derived cells, allowing for thorough screening of individual disease markers, and evaluating personalized neuroprotective drug activities [94].

Brain-on-chip models are developed to recapitulate brain tissue's physiological behavior and responses with the scope of testing the efficacy and toxicity of new drugs in well-defined and reproducible conditions. In 2015, Park *et al.* developed a microfluidic chip-based on 3D neurospheroids, which closely recapitulated the *in vivo* brain microenvironment by providing a constant dynamic flow (Fig. **5d**) [108]. Homogeneous neurospheroids were formed by imprinting a concave microwell array with the PDMS chip, resulting in spheroids with uniform size and 3D cytoarchitecture. An osmotic micropump system connected to the outlet generated a steady flow of medium at the interstitial level. The study showed that the flow rate has an inverse effect on spheroids' size as the size differentiation was significantly accelerated when the flow was reduced.

On the other hand, traditional mature molecules such as beta-III tubulin increased when the active interstitial flow was induced. By Integrating a 3D cytoarchitecture and interstitial flow, the developed chips mimicked the microenvironment of a normal and an AD diseased brain that can be used to investigate the effects of amyloid-β on neural tissue in a 3D environment. The toxicity of amyloid-β protein on neurospheroids was assessed under dynamic or static conditions for seven days. Neurospheroids cultured under dynamic (flow enabled) conditions demonstrated a larger size than those cultured under static (flow-disabled) conditions, while the amyloid-β protein reduced cell viability under static conditions. Moreover, under dynamic conditions, the study demonstrated that amyloid-β decreased the synapsing levels, permeating deeper into the neuronal tissue than in static assays [108].

Another 3D microfluidic AD model encompassing neurons, astrocytes, and microglia was recently developed by Park *et al.* This new platform exhibited most of AD's key characteristics, including p-tau accumulation, aggregation, and neuroinflammatory activity such as microglia recruitment and neurotoxic activity (including axonal cleavage) [109].

Furthermore, a recent study showed the application of a 3D microfluidic platform to cultivate PD patient-specific dopaminergic neurons. Compared to a 2D culture setting, the 3D approach revealed a significantly more robust endophenotype, thus providing the basis for the future development of personalized diseased models for drug discovery [110]. Recent innovations in chip-based technologies have made it possible to control neuronal connectivity *in vitro* for deciphering pathological and physiological processes within neuronal networks.

Primary brain tumors belong to a heterogeneous group of tumors that develop within the central nervous system (CNS), and approximately 75% of these tumors are gliomas [111]. These tumors are less prevalent than those which metastasize from different organs to the brain. Brain cancers give rise to a considerable mortality rate, and due to the restricted nature of the BBB, these cancers are remarkably challenging to treat. Organoids and microfluidic chips have recently been developed to recapitulate the microenvironment of a brain tumor *in vivo*. For instance, Fan *et al.* employed a polyethylene glycol diacrylate (PEGDA) hydrogel as a scaffold for drug screening to study gliomas. Glioblastoma cells derived from the U87 cell line formed 3D brain cancer tissue appropriately on the chip. The cells were examined for their responses to combinative treatment with Irinotecan and Pitavastatin. To do so, Fan and coworkers injected Pitavastatin and irinotecan into the cells, and the results demonstrated that this platform could be employed as a glioma chip model for release tests and drug screening [112]. To observe rat C6 glioma cell responses to colchicine anticancer drugs, Wang *et al.* developed a glioma-related microfluidic device with four parallel chambers. Interestingly, they witnessed fundamental variations in death rate and cell morphology over higher colchicine concentrations or extended treatment time. This study will not only be helpful in the development of the glioma-related anticancer drugs but also for glioma identification through the advancement of glial cell-based biosensors [113].

Recent studies exploit the capacities of suitable BBB models to represent the metastasis to the brain [114-116]. In particular, a 2016 research set apart a fluid-filled vascular chamber from gel-filled brain parenchyma with an ECM barrier coated with brain microvascular ECs on one side and astrocytes on the other; incorporation of flow in the vascular chamber and astrocytes were essential for a healthy BBB. Lung cancer, breast cancer, and malignant melanoma cell lines in the vascular chamber extravasated into the brain parenchyma and compromised the integrity of the BBB, while the liver cancer or primary brain cancer cell lines did not have any compromising impact on the BBB [117]. Equally significant, a 2019 study coupled a lung TME to a brain microenvironment and examined the metastasis of NSCLC to the brain. In this model, metastasizing NSCLC cells had to initially intravasate into the circulation, then travel to the brain compartment. Subsequently, these cells had to extravasate across the BBB and establish themselves in the brain parenchyma. The mentioned platform can adequately represent the metastatic capacity of various cell lines. This research demonstrated that the metastasis of NSCLC cell lines increases with the expression of the protein aldo–keto reductase family 1 B10 [118]. Additionally, Feng *et al.* observed the upregulation of MMP-2 and MMP-9 in metastatic locations in their study where they had a lung compartment upstream of a brain compartment with a functional BBB. Consequently, this MoC demonstrated an enhanced ability of metastatic subpopulations to compromise tight junctions and penetrate the BBB [119].

Terrell-Hall *et al.* developed a microfluidic chip model with two separate compartments (apical and basolateral) and an array of 3 μm pores [116]. HUVECs were cultured in the apical compartment, and these cells were exposed to fluid flow in both BTB and BBB models. In the BTB model, astrocytic cells were substituted with Met-1 metastatic murine breast cancer cells, whereas the BBB model was set up with CTX-TNA2 rat astrocytes in the basolateral compartment. Passive permeability studies of various compounds are reported to be comparable with *in vivo* permeabilities. Brown *et al.* further enhanced this model by coating the apical compartment with human fibronectin before culturing monolayers of a human BMVEC cell line (hCMEC/D3) which gave rise to a lumen-alike structure when exposed to a shear stress of 2.73 dyne/cm^2^ [120].

Organoids are miniaturized organ-like tissues generated from stem cells into a 3D structure showing realistic micro-anatomy. In neuroscience studies, organoids are implemented to generate a miniaturized brain comprising of neurons [121]. Efforts have been undertaken to bring about vascularized organoids using ECs within a clump of cells with an exhibition of critical junctional complex markers [122, 123]. Pham *et al.*, in their study, generated vascularized cerebral organoids by re-embedding promptly prepared organoids in matrigel droplets embedded with iPSC-derived endothelial cells [124]. In another similar approach, Bergmann *et al.* developed BBB organoids by promoting the accumulation of ECs, pericytes, and astrocytes under low-adhesion conditions [125]. Studying the vasculature and BBB surrounding organoids and spheroids can be more effective in mimicking the human brain microenvironment. Still, it also leads to the development of mature brain organoids through long-term culture and interaction among heterogeneous cells.

A combination of 3D printing and microfluidics platform has been used to improve the brain organoids model. Cho *et al.*, in their study about brain organogenesis, implemented a combination of a microfluidic platform with a decellularized human brain tissue-derived ECM to renovate brain-mimetic niches to conduct neural and glial differentiation [126]. 3D-bioprinting technology has also been utilized to generate a microfluidic-based vascularized system to provide defined spatial sites, and hence interactions among neural organoid models and vascular tissue [127]. Qian *et al.*, in their research, suggested a spinning millifluidic system using human iPSCs to generate forebrain-specific organoid systems which simulate the fundamental aspects of human cortex growth. They also modeled Zika virus (ZIKV) exposure with their proposed platform. Quantifiable investigations illustrated that either African or Asian ZIKV strains infect progenitors of the brain effectively [128]. Moreover, the infection caused by ZIKV resulted in higher cell death and lower proliferation which led to reduced neuronal cell-layer volume [128].

Novel corona-virus 2 (SARS-CoV-2) prompted various research groups to utilize organ-on-chip (OoC) platforms [129-131], and organoids [132] to further investigate the disease. Pellegrini *et al.* studied SARS-CoV-2 neurotropism by implementing hPSC-derived brain organoids [132]. They infected the organoids by spiking SARS-CoV-2 pseudovirus and live virus, which led to the viral tropism for choroid plexus epithelial cells expressing apolipoprotein and ACE2. The infection of choroid plexus epithelium may undermine the B-CSF-B barrier, followed by neurological problems due to the pro-inflammatory molecules and leakage. However, a minor infection was witnessed for neurons or glia. Ramani *et al.*, in another fascinating study, demonstrated that within two days of exposure to SARS-CoV-2, the virus enters the 3D human brain organoid models and consequently targets the neurons [133].

The complexity of cell communication in the brain demands a considerable research effort to recreate the heterogenicity of cellular interactions in microfluidic devices. Recent advancement in BBB chips and brain-on-a-chip technologies, including the integration of biosensors, has significantly expanded the capabilities of these models as tools for drug development and discovery but has also improved their translational significance and reliability in recapitulating the essential biological functions and pathological responses of their *in vivo* counterparts. These unique features enable their use to unravel crucial mechanistic aspects of the NVU in both diseased and healthy conditions.

### 4.2 Application of Vascular on a Chip for Pathogenic Condition

Normal physiological processes in the microvascular system, such as angiogenesis, juxtacrine and paracrine interactions between ECs and perivascular cells, and inflammation are essential for maintaining a healthy body and stable circulation. Any dysfunctions to these processes result in the development of different disease conditions. Vascular-on-a-chip microfluidic platforms are considered one of the most promising models to study human diseases, specifically vascular pathophysiological conditions, including endothelial dysfunction, inflammations, thrombosis, tumor angiogenesis, and cancer metastasis.

#### 4.2.1 Endothelial Dysfunction

Endothelial dysfunction is a critical physiological process that can lead to thrombosis, atherosclerosis, or inflammatory diseases [134-137]. Multiple studies have been conducted to elucidate the underlying mechanisms of endothelial dysfunction and related disorders [19, 138-140]. Under normal conditions, circulating blood components do not adhere to the intact vessel walls but interact with the injured ones in response to endothelial activation. The activated endothelium releases the pro-inflammatory content of their secretory granules (Weibel‐Palade bodies - WPBs), including Von Willebrand factor (VWf), Tissue Factor, Endothelin-1 tumour-necrosis factor (TNF), interleukin-1 (IL-1), *etc.* At the same time, surface adhesion molecule expression is increased, facilitating the binding and activation of platelets and leukocytes [141], thus promoting blood coagulation and vascular inflammation. Engineered vascular models have been successfully utilized to investigate these crucial processes, especially the role of hemodynamic and biochemical factors during inflammation and initiation of thrombosis.

#### 4.2.2 Inflammation

Microfluidic devices are an innovative platform to study endothelium-mediated inflammatory processes such as leukocyte chemotaxis, rolling, adhesion, extravasation, and transmigration [142, 143]. In a reported study, TNF-α was used to induce the expression of leukocyte adhesion molecules within engineered microvessels to trigger neutrophil rolling and adherence to the activated endothelium [144]. Moreover, creating a cytokine gradient external to the synthetic microvessel induced diapedesis and chemotaxis [144]. Microfluidic models have also enabled the understanding of T-cell and neutrophil chemotaxis patterns at the level of single cells [145, 146]. Using patient samples, these models have also been employed to investigate specific disease states such as acute respiratory distress syndrome and trauma/burns [147, 148].

#### 4.2.3 Thrombosis

In cases of severe inflammation, such as severe sepsis or massive trauma, the interrelationship of the inflammatory and coagulation pathways reaches its peak where broad microvascular thrombi can result from dysregulation of the inflammatory cascades [149]. The initiation of thrombosis by adhesion and aggregation of platelets largely depends on blood flow and shear stress at local sites [150]. One of the advantages of microfluidic devices over traditional 2D cultures is the ability to study the role of flow and three-dimensional vessel geometries on thrombotic progression [150]. In a study by Westein *et al.*, a microfluidic device with stenotic areas integrated into flow channels showed that platelets interacted with VWF to aggregate in poststenotic areas [151]. The study also revealed that high shear stress levels induce endothelial cells to release more VWF that further increases platelet adhesion [151].

In addition, Zheng *et al.* built 3D microvessels to study the initial steps of thrombosis. In their study, the investigators highlighted the importance of the endothelial activation steps and vessel geometry in blood-vessel interactions [38]. They showed that activated endothelium expressed VWF fibers on their surface which bound platelets, with 3D networks of VWF fibers forming near vessel bifurcations and junctions. A subsequent study showed the effects of vessel diameter, 3D vessel geometry, shear stress, and flow acceleration on the 3D assembly of the shear-sensitive protein VWF, which formed complex interactions with blood cells, including platelets, leukocytes, and red blood cells [38].

**Fig. (7):**
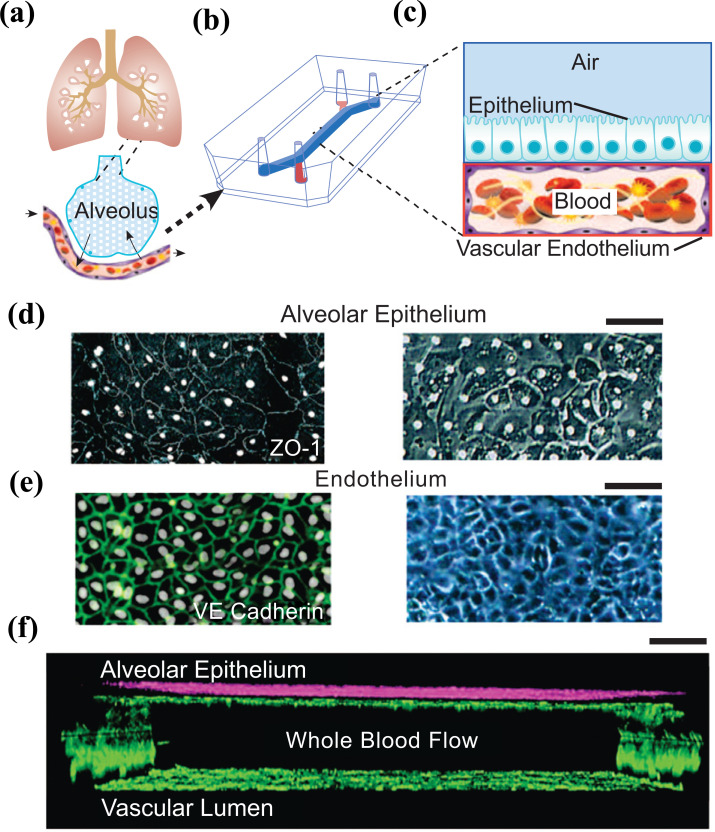
**a**) conceptual schematic of the human lung showing that the alveoli interact with the neighboring blood vessels during hemostasis or pulmonary dysfunction. **b**) Engineering drawing of the microdevice containing two PDMS compartments separated by a thin, porous membrane that reproduces the microarchitecture of the alveolar-capillary interface. **c**) Graphic illustration showing the top compartment (1 mm wide and 1 mm tall) is cultured with human primary alveolar epithelial cells and the entire bottom chamber (1 mm wide and 250 µm tall) lined with human endothelial cells forming a lumen. Whole blood is perfused through the bottom chamber and thrombus formation is visualized using fluorescence microscopy from the bottom. **d**) Micrograph of human lung alveolar epithelial cells (ZO1, left; brightfield, right; Scale bar, 50 µm) and **e**) Vascular endothelial cells (VE-cadherin, left; brightfield, right, Scale bar, 50 µm) **f**) Sideview of confocal micrographs showing junctional structures, after twelve days of co-culture, of a single layer of the primary alveolar epithelium at the top chamber (purple, stained with E-cadherin) and endothelial monolayers covering the entire surface of the lower chamber (green, stained with VE-cadherin), through which blood perfusion takes place. Scale bar: 100 µm. Reproduced with permission from reference [152].

Recently, a microfluidic chip was designed to study pulmonary thrombosis (Fig. **7**). The device comprises two PDMS microchannels, separated by a porous, thin, and flexible PDMS membrane to reproduce the microarchitecture of the alveolar-capillary interface. Human blood was used instead of a cell culture medium to mimic *in vivo* conditions and evaluate antithrombotic drugs [152]. They demonstrated that when the endothelium was stimulated with TNF-α before initiating blood flow, the inflamed vascular endothelium promoted rapid platelet recruitment and thrombus formation as observed within inflamed microvessels *in vivo*. Then, the effectiveness of the drug candidate parmodulin-2 (PM2), a potent protease-activated receptor-1 inhibitor with antithrombotic and vascular protective effects, was assessed in the model. The results showed that PM2 treatment significantly reduced platelet-endothelial binding compared to controls [152].

In summary, the studies mentioned above clearly highlight the capability and potential usefulness of vascular-on-a-chip models to reproduce a large variety of hemodynamic and blood hemostatic problems and aid in the design of antiplatelet or anticoagulant drug treatments.

#### 4.2.4 Angiogenesis/Vasculogenesis and Tumor Biology

Cancer research is one of the most significant areas that could benefit from developing a realistic *in vitro* model of vascular functions. Cancer growth is linked to the interactions between tumor cells and different tissue microenvironments [153]. Tumors promote angiogenesis and drive the creation of tumor vessels. These newly formed vessels may be highly abnormal, with disorganized vasculature networks, dysfunctional or absent perivascular cells, lymphatics, and increased vascular permeability. All these factors are thought to promote tumor intravasation and metastasis. Therefore, a complete understanding of the processes involved in tumor angiogenesis and metastasis is essential for developing more effective treatments [154]. Investigators have used microfluidic devices to study tumor angiogenesis in which tumor cells were co-cultured with the endothelium [154]. For example, Kim *et al.* fabricated a microfluidic chip to investigate cancer angiogenesis. In this platform, fibrin gel was perfused into the central channel as a matrix for endothelial sprouting. Cancer cells were mixed with fibrin gel and introduced into the side channel, and ECs were attached to the fibrin gel wall. The investigators found that coculture of endothelial cells with cancer cells within the microvessel model led to aberrant angiogenesis when compared against parallel systems where the endothelium was instead cocultured with fibroblasts instead of tumor cells [56]. In another work conducted by Buchanan *et al.*, alterations in vascular permeability were observed when tumor cells were cultured with endothelial cells. The investigators also determined that increased shear stress is associated with decreased permeability and downregulation of tumor angiogenic genes, suggesting that the low-flow state seen in many tumor vessels may contribute to tumor angiogenesis [155].

## CONCLUSION

Recent advances in microfluidic technologies allow the development of novel *in vitro* models of vascular tissue. This comprehensive review assesses the latest microfluidic vascular models in drug development and pathophysiology studies. The examples provided herein support the notion that microfluidic technology can facilitate current vascular research and discusses areas of investigation that are not easily accessible with more traditional setups and techniques. These vascular platforms have been designed to incorporate various techniques, including microvessel patterning methods and angiogenesis/vasculogenesis-based approaches, which are discussed thoroughly in this review. The latest achievements in developing microfluidic vascular organs, including lung, heart, and brain microvasculature were also highlighted and discussed. Of note is the importance of these organ-on-a-chip models in the field of precision medicine. Because patients suffering from the same illness may experience a different response to treatment, creating patient-specific vascular models could help develop more effective and safer individualized therapies. It is also possible to envision at some point the development of humanized multi-organ-on-a-chip platforms where various vascular organs are integrated into a single chip. Thus, allowing not only to reproduce the specific characteristics of the desired organs but also to mimic organ-organ interactions.

It is important to mention that there are several challenges yet to be addressed concerning organ-on-a-chip devices. Standardized techniques and materials are required to boost the development of technology for large-scale fabrication and distribution. For this reason, the application of microfluidic technology in laboratory settings has been relatively sparse and limited in scope. Almost all studies reported in this review are proof-of-principle models that require specific skill sets, dedicated personal effort, and ad hoc intervention by the researcher pioneering the technology, which is still in its infancy. Moreover, due to the lack of standardization, and the use of different methods and components (both biological and synthetic), the evaluation and cross-comparison of these models to assess their translational relevance is quite complicated.

The principle of dose-response may bring about a valuable investigative role in evaluating the possible biological functions of a feasible drug compound and its therapeutic implementations in vascularized *in vitro* models. From this perspective, it is important for the reader to familiarize with the concept of Hormesis which refers to adaptive responses of biological systems to moderate environmental or self-imposed challenges. Through this process, the above-mentioned biological system can improve its functionality and/or become more resilient to more severe challenges [156]. One such example of hormetic response is the effect of resveratrol on human cancerous cell lines [157-159]. It was observed that low doses of resveratrol had a protective effect however adverse outcomes resulted from high doses leading to disease progression. It is important to note that hermetic responses vary by the type of model used, wherein there is a lack of translatability between cell culture, animal, and humans. As a result, microfluidic models can be crucial for investigating hormetic reactions since they allow for the study of human related responses in a multicellular dynamic co-culture model.

## LIST OF ABBREVIATIONS

BBB: Blood-brain Barrier
BTB: Blood-tumor Barrier
BCSFB: Blood-cerebrospinal Fluid Barrier
CNS: Central Nervous System
NVU: Neurovascular Unit
ECs: Endothelial Cells
SMCs: Smooth Muscle Cells
PDMS: Polydimethylsiloxane
HUVECs: Human Umbilical Vein Endothelial Cells
ECM: Extracellular Matrix
PEG: Polyethylene Glycol
GelMA: Gelatin-methacryloyl
PEGTA: Polyethylene Glycoltetra-acrylate
PEGDA: Polyethylene Glycol Diacrylate
VEGF: Vascular Endothelial Growth Factor
BFGF: Basic Fibroblast Growth Factor
BM-hMSCs: Human Mesenchymal Stem Cells Derived from Bone Marrow Stroma
LOC: Lung-on-a-Chip
TNFα: Tumor Necrosis Factor α
ICAM-1: Intercellular Adhesion Molecule 1
PS: Phosphatidylserine
COPD: Chronic Obstructive Pulmonary Disease
JAK: Janus Kinases
TEM: Transmission Electron Microscopy
PLGA: Polylactic-co-glycolic Acid
HFL1: Human Fetal Lung Fibroblasts
EGFR: Epithelial Growth Factor Receptor NPs Nanoparticles CMs Cardiomyocytes
CVDs: Cardiovascular Diseases
HoC: Heart-on-chip
MoC: Multi-organ-on-a-chip
OoC: Organ-on-a-chip
PC: Polycarbonate
iPSC: Induced Pluripotent Stem Cell
hESCs: Human Embryonic Stem Cells
P-gp: P-glycoprotein
BMECs: Brain Microvascular Endothelial Cells
ZIKV: Zika Virus
AD: Alzheimer's Disease
VWf: Von Willebrand Factor
TNF: Tumor-necrosis Factor
IL-1: Interleukin-1
PM2: Parmodulin-2
MPS: Mononuclear Phagocyte System
TEER: Trans-endothelial Electrical Resistance
